# A Comparative Study of E-Beam Deposited Gate Dielectrics on Channel Width-Dependent Performance and Reliability of *a*-IGZO Thin-Film Transistors

**DOI:** 10.3390/ma11122502

**Published:** 2018-12-09

**Authors:** Gwomei Wu, Anup K. Sahoo, Dave W. Chen, J. W. Chang

**Affiliations:** 1 Institute of Electro-Optical Engineering, Chang Gung University, Taoyuan 333, Taiwan; anup140387@gmail.com; 2 Chang Gung Memorial Hospital, Keelung 204, Taiwan; mr5181@cgmh.org.tw (D.W.C.); flyinwei@gmail.com (J.W.C.)

**Keywords:** *a*-IGZO, thin-film transistor, e-beam, gate dielectric, reliability

## Abstract

A comparative study on the effects of e-beam deposited gate dielectrics for amorphous indium gallium zinc oxide (*a*-IGZO) thin-film transistors (TFTs) has been carried out using SiO_2_, Si_3_N_4_, and Ta_2_O_5_ dielectric materials. The channel width dependent device electrical performances were investigated using three different sizes of 500 μm, 1000 μm, and 1500 μm. The reliability characteristics were revealed by the threshold voltage variation and drain current variation under positive bias stress. The e-beam deposited high-k dielectric Ta_2_O_5_ exhibited the highest stability at the stress voltage of 3 V for 1000 s due to its high capacitance density at 34.1 nF/cm^2^. The threshold voltage variation along the channel width decreased from SiO_2_, then Si_3_N_4_, to Ta_2_O_5_, because of the increased insulating property and density of capacitance. The SiO_2_-based *a*-IGZO TFT achieved a high field effect mobility of 27.9 cm^2^/V·s and on–off current ratio > 10^7^ at the lower channel width of 500 μm. The gate leakage current also decreased with increasing the channel width/length ratio. In addition, the SiO_2_ gate dielectric-based *a*-IGZO TFT could be a faster device, whereas the Ta_2_O_5_ gate dielectric would be a good candidate for a higher reliability component with adequate surface treatment.

## 1. Introduction

Recent studies have been focused on several transparent conducting oxides (TCO), such as zinc oxide (ZnO), indium zinc oxide (IZO), amorphous zinc tin oxide (*a*-ZTO) and amorphous indium gallium zinc oxide (*a*-IGZO) [1,2,3]. These materials showed advantages, such as an easy film coating process, better surface morphology, and electrical stability during operation. Among them, *a*-IGZO has been proven to be a promising material because of its high mobility and transparency [4]. It exhibited a smooth surface quality when deposited at room temperature [5]. Therefore, this oxide material has been investigated for the active channel layers in thin-film transistors (TFTs). In addition, *a*-IGZO TFTs can be fabricated on silicon wafer, glass, and also flexible organic substrates. The prevailing amorphous silicon (*a*-Si:H) exhibits a low carrier mobility (0.5–1 cm^2^/V·s), while the polycrystalline silicon (poly-Si) requires high-temperature fabrication processes (>500 °C) [6,7]. However, recent studies have found that the performance of TFTs is very much dependent upon the gate dielectric material and its deposition method [8].

Silicon dioxide (SiO_2_) has been used as the gate dielectric in *a*-IGZO TFTs because of its thermal stability and smooth surface morphology. Most of the reported results achieved mobility for smaller channel dimension-based *a*-IGZO TFTs < 20 cm^2^/V·s, using various coating methods, such as thermal growth, sputtering, and plasma-enhanced chemical vapor deposition (PECVD) [9,10,11,12]. On the other hand, *a*-IGZO TFTs with a bigger channel size have been studied using e-beam deposited SiO_2_ gate dielectric. The electrical performances could be significantly improved after employing plasma treatment on the gate dielectric [13].The atomic layer deposited (ALD) SiO_2_ gate dielectric was also reported on *a*-IGZO TFTs that could maintain a good stability under a negative gate bias stress, while exhibiting very high field-effect mobility without any post-annealing [14].

In the literature, several high-k gate dielectrics have been introduced to increase the mobility and reliability of *a*-IGZO TFTs. High-k gate dielectrics can provide a high gate capacitance and low leakage current with an equivalent oxide thickness, and the drivability can be further improved. For example, Chiu et al. reported a room-temperature deposited *a*-IGZO channel with tantalum pentoxide (Ta_2_O_5_, high-k ~ 29) exhibited field-effect mobility of 61.5 cm^2^/V·s [15]. Qian et al. achieved a mobility of 30.1 cm^2^/V·s for *a*-IGZO TFTs using Ta_2_O_5_ dielectricwithlanthanum (La) incorporation [16]. However, critical issues related to the threshold voltage variation along the channel width, bias-stress instability, and device reliability are still challenging, which can seriously influence the practical applications of *a*-IGZO TFTs. There are a number of reports on *a*-IGZO TFTs with a comparative study of different dielectric materials prepared by the same coating technology. Lee et al. performed a study on the electrical characteristics and device instabilities in *a*-IGZO TFTs with four different high-k gate dielectrics (Al_2_O_3_, HfO_2_, Ta_2_O_5_, and ZrO_2_), deposited with the radio frequency (RF) sputtering method [8]. They concluded that higher k dielectric ZrO_2_ is the most preferable candidate in terms of bias stress, mobility, or current on–off ratio. The highest achieved mobility was 10.2 cm^2^/V·s for high-k dielectric ZrO_2_. Lin et al. investigated *a*-IGZO TFTs using different high-k gate dielectric materials, such as silicon nitride (Si_3_N_4_) and aluminum oxide (Al_2_O_3_), at a low temperature process (<300 °C) and compared them with low-temperature SiO_2_ [17]. However, they achieved a mobility <10 cm^2^/V·s and had a large variation of electrical properties in a stressed situation. Therefore, there are still some obstacles to this oxide being useful in real applications, especially with a combination of different channel widths and different gate dielectrics using different deposition methods. The performances of *a*-IGZO TFTs with different combinations of gate dielectric and channel width have not been clearly investigated. The TFT performances could be affected with conjugate effects because of the variation in electric field, surface roughness, and insulating property. In addition, the threshold voltage variation along different channel widths with e-beam deposited gate dielectric remains questionable. It is, thus, very important to investigate the reliability characteristics with different dielectrics. 

In this report, the comparative performances of *a*-IGZO TFTs were studied with e-beam deposited gate dielectrics of SiO_2_, Si_3_N_4_, and Ta_2_O_5_. A distribution of threshold voltage was carried out with three different channel width sizes for each gate dielectric. The influence by combination of channel width and gate dielectric on *a*-IGZO TFTs electrical properties was thus investigated. The reliability performances with a respective variation in threshold voltage and drain current of *a*-IGZO TFTs under the stress voltage of 3 V for 1000 s measurement were evaluated. The mechanism explained the variation in threshold voltage and drain current for the various dielectric systems. In addition, the threshold voltage variation along the 500–1500 μm channel width decreased from SiO_2_, then Si_3_N_4_, to Ta_2_O_5_, because of the increased insulating property and density of capacitance. The e-beam deposited high-k dielectric Ta_2_O_5_ exhibited the highest stability due to its high capacitance density. Moreover, this study classified that Ta_2_O_5_ gate dielectric would be a good candidate for long-term reliability components.

## 2. Materials and Methods

**Figure 1:**
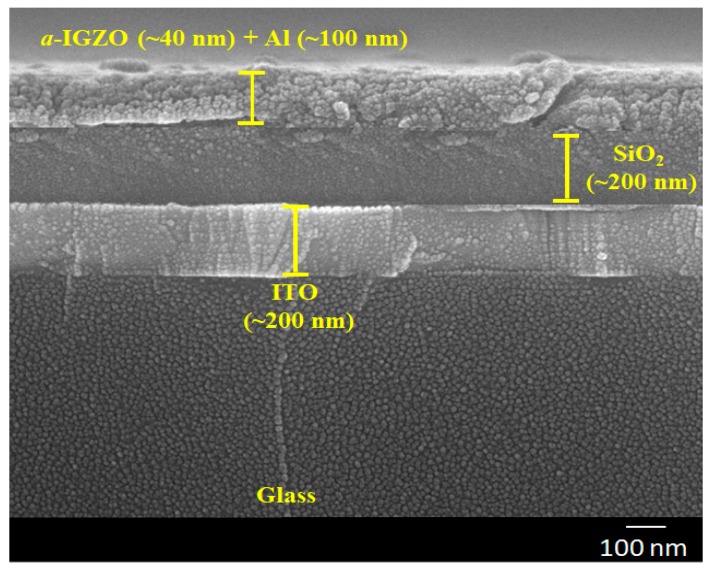
A cross-section SEM image for the multiple films on glass substrate.

**Figure 2:**
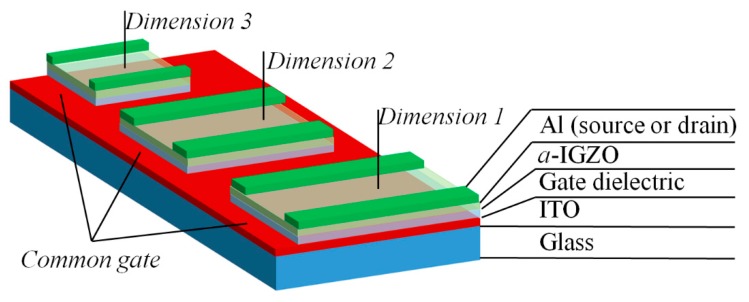
A schematic representation of the multiple dimension device structure.

In the experiments, sputter-deposited 100nm indium tin oxide (ITO)-coated glass, with a sheet resistance of 15 ohm/sq, was used as the substrates. The substrates were washed in an ultrasonic bath with acetone, isopropyl alcohol, and de-ionized water for 15 min in each step. The substrates were then dried by N_2_ gun and a hot plate at 120 °C for 1 h before use. After that, a 1 mm × 2 cm area size from one side was covered using a vacuum tape with a shadow mask on the common bottom gate of ITO. The system was designed to make patterns for gate dielectrics and the *a*-IGZO layer for the relatively large channels. Samples with different types of gate dielectric materials were deposited by an e-beam evaporator system using an SiO_2_, Si_3_N_4_, and Ta_2_O_5_ source, respectively. Before the evaporation of each of the three gate dielectric materials, the e-beam chamber vacuum base pressure was about 8 × 10^−6^ Torr (10.6 × 10^−^^4^ Pa). However, the chamber pressure during the evaporation increased to the order of 2–4 × 10^−5^ Torr (2.6–5.3 × 10^−^^3^ Pa). The deposition rate was about 1–10 nm/s. The temperature of the e-beam chamber was varied at 23–30 °C, 23–40 °C, and 23–45 °C during the film deposition of SiO_2_, Si_3_N_4_, and Ta_2_O_5_, respectively. This was likely caused by the different melting points of the materials (SiO_2_ ~ 1423 °C, Si_3_N_4_ ~ 1700 °C, and Ta_2_O_5_ ~ 1875 °C) due to the heating effect of the e-beam sources [18,19]. The deposition current was also varied for SiO_2_, Si_3_N_4_, and Ta_2_O_5_ at about 5–6, 16–17, and 20–21 amps (A), respectively, at a fixed voltage of 5 kV. The thickness of each gate dielectric layer was kept the same at ~200 nm. There was no intentional substrate heating for all cases. Additionally, all dielectric layers were deposited by the e-beam evaporator on p+ Si at the same instant to evaluate the density of capacitance with fabricated metal-insulator–semiconductor (MIS) capacitors. Then, *a*-IGZO ~ 40 nm films were deposited on the bi-layers of SiO_2_/ITO glass, Si_3_N_4_/ITO glass, and Ta_2_O_5_/ITO glass by RF sputtering. The deposition power was fixed at 70 W and the pressure was about 3 m Torr (0.4 Pa). The Ar/O_2_ inlet gas ratio was 50:1 for all the different gate dielectric-based samples. All the deposition conditions were kept the same at all times to avoid any other uncertain effect. After that, 100 nm Al film was coated by a thermal evaporator system and then patterned to form the source and drain of the thin-film transistor, followed by photolithography and a lift process. A cross-section scanning electron microscope (SEM, Hitachi S-5000, Tokyo, Japan) image is presented here to qualify the thickness of multiple films in Figure 1. A layer-by-layer structure can be observed. The channel width was varied at 500µm, 1000 µm, and 1500 µm, while the channel length was fixed at 200 µm. Figure 2 shows a schematic representation of the final device structure. In addition, *a*-IGZO films were grown on clean bare glass to be used for an evaluation of the physical characteristics of the surface roughness by atomic force microscopy (AFM, Park System, XE-70, Santa Clara, CA, USA) and the optical transmittance spectra by a UV spectrometer (Jasco, Tokyo, Japan, ISN-723). The three different MIS capacitors were then measured using a 4284A precision LCR (inductance, capacitance, and resistance) meter at constant frequency of 1 MHz. The TFT device electrical properties, such as transfer characteristics, output characteristics, and bias stress characteristics, were evaluated with a semiconductor parameter analyzer (B1500A, Agilent, Santa Clara, CA, USA).

## 3. Results and Discussion

**Figure 3:**
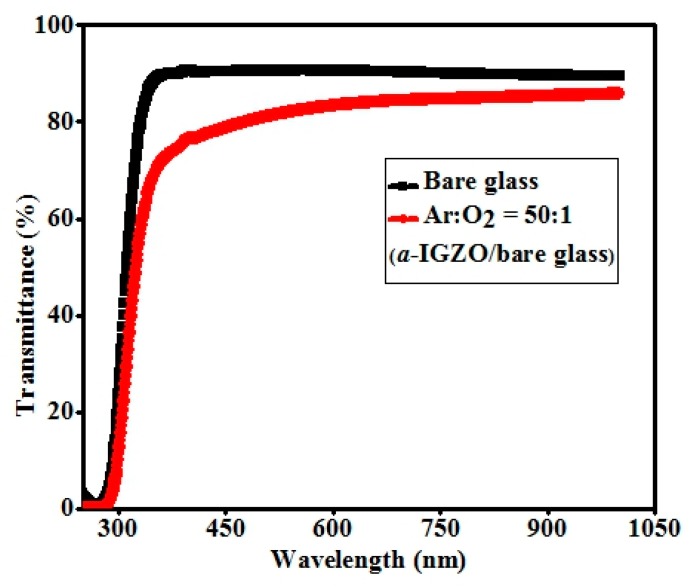
Optical transmission spectra of RF sputtered as-deposited *a*-IGZO films on clean bare glass.

The optical transmittance spectra for the as-deposited *a*-IGZO films on clean bare glass are shown in Figure 3 with the optimized argon to oxygen flow rate 50:1. The average transmittance of *a*-IGZO films exhibited above 80% in the visible and near-infrared range (VNIR, wavelength range 400 nm to 1000 nm). The band gap energy of the *a*-IGZO film could be derived using a plotting method [20] The band gap energy of films was thus obtained at about 3.49 eV by extrapolating the straight-line portion of (*αhν*)^2^ vs. *hν* plots to the energy axis. Here, *h* and *ν* represent for the Planck constant and the radiation frequency.

**Figure 4:**
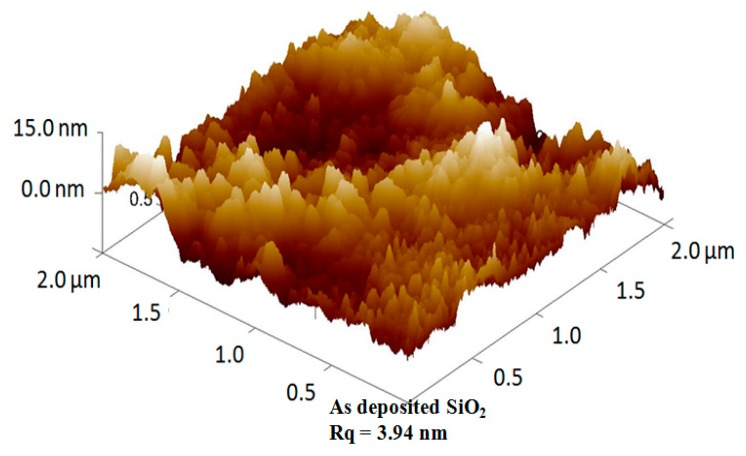
Atomic force microscopy (AFM) image of the e-beam deposited SiO_2_ film on indium tin oxide (ITO) glass substrate.

The top surface morphology of the e-beam deposited dielectric layers were investigated using AFM image analysis and the as-deposited SiO_2_ film on ITO glass substrate image is hereby shown in Figure 4. The surface roughness (RMS) Rq values of the e-beam deposited SiO_2_, Si_3_N_4_, and Ta_2_O_5_ were 3.94 nm, 4.06 nm, and 4.11 nm, respectively. The high-k dielectric Si_3_N_4_ and Ta_2_O_5_ had a slightly higher surface roughness than the SiO_2_. This has been attributed to the higher heating effect by the larger temperature variation range, for Ta_2_O_5_ ~ 22 °C, Si_3_N_4_ ~ 17 °C, and SiO_2_ only ~7 °C, and also possibly an enhanced pressure variation during the film deposition process.

**Figure 5:**
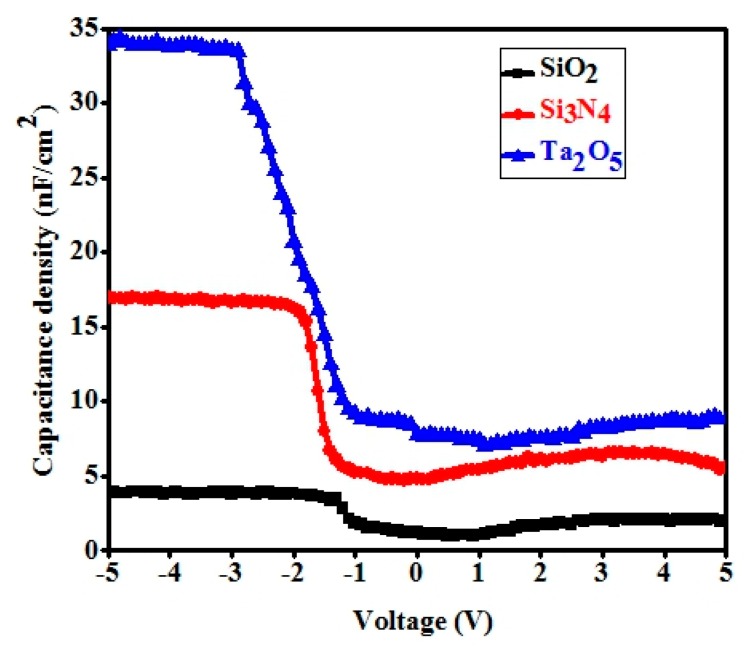
Capacitance density vs. voltage characteristics of the metal-insulator–semiconductor (MIS) capacitor with gate dielectric SiO_2_, Si_3_N_4_, and Ta_2_O_5_ deposited on p + Si wafer.

The capacitance variation of the e-beam deposited dielectric materials was measured on p^+^ Si wafer with the MIS structures. The capacitance vs. applied voltage characteristics with a sweep voltage of −5 V to +5 V are shown in Figure 5. The maximum capacitance density (*C*_ox_)was achieved at the accumulation region at about 4 nF/cm^2^, 16.9 nF/cm^2^, and 34.1 nF/cm^2^ for the corresponding SiO_2_, Si_3_N_4_, and Ta_2_O_5_ gate dielectric materials. The e-beam deposited Ta_2_O_5_ gate dielectric material showed a much higher insulating property compared to SiO_2_ and Si_3_N_4_. Nevertheless, much higher capacitance density dielectrics can be further explored by multiple oxides using more sophisticated deposition techniques. 

**Figure 6:**
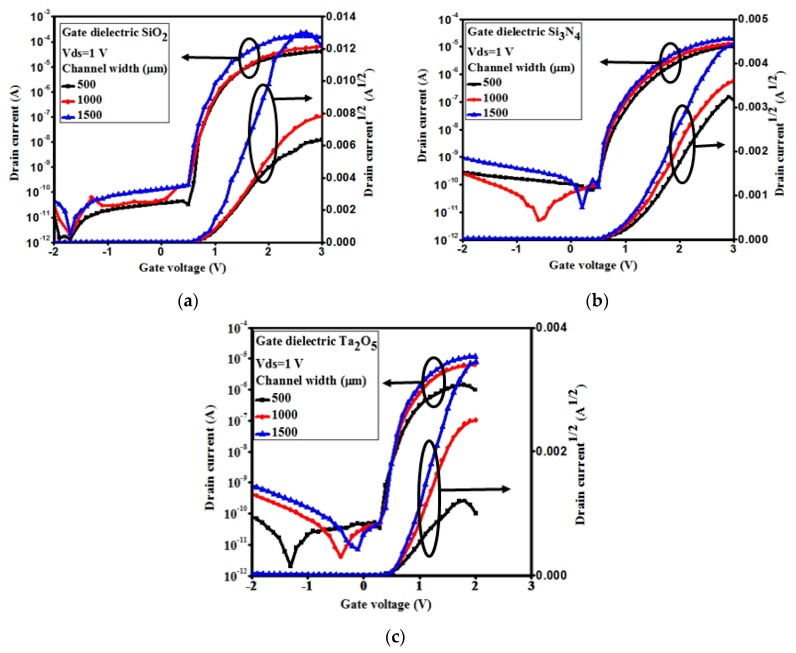
Typical transfer characteristics of the *a*-IGZO-based thin-film transistors (TFTs) with gate dielectric (**a**) SiO_2_, (**b**) Si_3_N_4_, and (**c**) Ta_2_O_5_ at different channel widths of 500, 1000, and 1500 µm. The drain voltage was 1 V, and the right-hand side graph shows the characteristics of I_d_^1/2^ vs. voltage to determine the threshold voltages.

The channel width dependent transfer characteristics of the *a*-IGZO TFTs with the different e-beam deposited gate dielectrics of SiO_2_, Si_3_N_4_, and Ta_2_O_5_ are shown in Figure 6. The characteristics could have also been represented for the channel size width of 500 μm, 1000 μm, and 1500 μm, respectively, at the fixed channel length of 200 μm. The transfer characteristics of *a*-IGZO TFTs were significantly changed with the various gate dielectric materials as well as with the various channel widths in TFTs. Nevertheless, the performances of the nine different types of TFTs could be compared by evaluating their basic performance parameters, such as field effect mobility (*µ*_fet_), threshold voltage(*V*_th_), sub-threshold swing voltage (*SS*), and on-current to the off-current ratio (*I*_on_/*I*_off_).

Table 1, Table 2 and Table 3 summarize the evaluated TFT performance parameters for each of the gate dielectric material systems used in this study. The drain to source voltage *V*_ds_ keeps a constant at 1 V for all samples. The capacitance density data have been derived for the three dielectric materials. The field effect mobility (*µ*_fet_) at the saturated regime has been derived by the following equation:

(1) $${µ}_{\mathrm{fet}}=\frac{{\left(\frac{d{\sqrt{I}}_{\mathrm{ds}}}{dV_{\mathrm{gs}}}\right)}^{2}}{\frac{1}{2}\;C_{\mathrm{ox}}\cdot \left(\frac{W}{L}\right)\cdot V_{\mathrm{ds}}},$$

where *W* is the width and *L* is the length of TFT channel.The evaluated mobility *µ*_fet_ using the three gate-insulting materials SiO_2_, Si_3_N_4_, and Ta_2_O_5_ is 27.9 cm^2^/V·s, 20.6 cm^2^/V·s, and 12.1 cm^2^/V·s, respectively, at the fixed channel width of 500 μm. The mobility has been extracted at agate voltage of 1.4~1.5 V. The evaluated data become 21.6 cm^2^/V·s, 13.5 cm^2^/V·s, and 6.4 cm^2^/V·s, respectively, at the channel width of 1000 μm and they are 13.5 cm^2^/V·s, 8.8 cm^2^/V·s, and 4.7 cm^2^/V·s at the fixed channel width of 1500 μm. It was immediately noted that the lower values in mobility were associated with the high-k dielectric Si_3_N_4_ and Ta_2_O_5_-based *a*-IGZO TFTs. That was likely caused by the higher surface roughness of the as-deposited gate dielectric Si_3_N_4_ (Rq 4.06 nm) and Ta_2_O_5_ (Rq 4.11 nm) films. Nevertheless, the performances of *a*-IGZO TFTs could be further enhanced with special treatment by high temperature annealing or plasma surface modification. In discussion, the mobility was much more dependent on the surface roughness of the e-beam deposited gate dielectric, rather than the insulating properties of film. It will be more necessary for a good high temperature annealing process to reduce the surface roughness before their use in *a*-IGZO TFTs application. On the other hand, the mobility of TFTs also depends on the channel width of TFTs for all dielectrics used in the study. The higher mobility can be achieved at lower channel dimension-based TFTs. The decreasing trend was observed for the three gate dielectric material systems.

A high speed and low power operation are important for TFT applications. This characteristic could be evaluated by the sub-threshold swing voltage (*SS*) of *a*-IGZO TFTs. A smaller *SS* value suggests device stability, higher mobility, and lower interfacial trap charge density. The evaluated sub-threshold swing voltage *SS* that used the three gate insulting dielectrics SiO_2_, Si_3_N_4_, and Ta_2_O_5_ are 0.11 V/dec, 0.15 V/dec, and 0.14 V/dec, respectively, at the fixed channel width of 500 μm. The *SS* data become 0.11 V/dec, 0.13 V/dec, and 0.11 V/dec, respectively, at the channel width of 1000 μm, and 0.10 V/dec, 0.11 V/dec, and 0.10 V/dec at the channel width of 1500 μm. It was evidenced that the *SS* values are shown lower for the SiO_2_-based *a*-IGZO TFTs when compared with the Si_3_N_4_- and Ta_2_O_5_-based *a*-IGZO TFTs. The tendencies are similarly shown from the lower to higher sizes devices. This result suggested a better interface region in the SiO_2_ gate dielectric and *a*-IGZO channel system. However, the variation became much smaller for the higher size devices with the channel width of 1500 μm. The interfacial region could be enhanced with a trap charge density by introducing high-k dielectric Si_3_N_4_ and Ta_2_O_5_. Additionally, the interfacial characteristics could be improved with bigger channel width sizes for high-k-based gate dielectrics.

The comparative performances of *a*-IGZO TFTs with the e-beam deposited gate dielectric SiO_2_, Si_3_N_4_, and Ta_2_O_5_ along the different channel width sizes were further investigated by evaluating the on-current to the off-current ratio. A significant variation was observed among all the *a*-IGZO TFTs with different gate dielectrics as well as different channel width sizes. The *I*_on_/*I*_off_ ratios were 2.9 × 10^7^, 1.5 × 10^5^, and 7.1 × 10^5^ for the SiO_2_, Si_3_N_4_, and Ta_2_O_5_ TFT devices, respectively, at the fixed channel width of 500 μm. They became 4.6 × 10^7^, 2.1 × 10^6^, and 2.1 × 10^5^, respectively, at the channel width of 1000 μm, and 8.1 × 10^7^, 7.1 × 10^6^, and 1.6 × 10^6^ at the channel width of 1500 μm. The *I*_on_/*I*_off_ values were generally increased with the increasing channel width sizes. However, they were eventually decreased for the high-k gate dielectric Si_3_N_4_ and Ta_2_O_5_ compared to the SiO_2_ samples. The noticeable variation in the off-current level and on-current level occurred. The lower off-current level or gate leakage current was achieved with an increased capacitance density corresponding to the gate dielectrics, whereas the opposite phenomena were observed for the on-current level situation. The results clearly indicated that the as-deposited e-beam high-k Si_3_N_4_ and Ta_2_O_5_ films could not improve the performances of *a*-IGZO TFTs because of the higher surface roughness. An improved annealing process and/or plasma treatment is required to reduce the surface roughness. On the other hand, the on–off current level increased with increased channel width, because of the increase in parasitic resistance between the channel surface and the source–drain electrode. Still, the distribution of the off-current level along the different channel width was increased for the gate dielectric SiO_2_ (10^−12^–10^−11^), Si_3_N_4_ (10^−11^–10^−9^) to Ta_2_O_5_ (10^−12^–10^−9^). The wider scattering of the off-current level was likely caused by the increased surface roughness of Si_3_N_4_ and Ta_2_O_5_.

**Figure 7:**
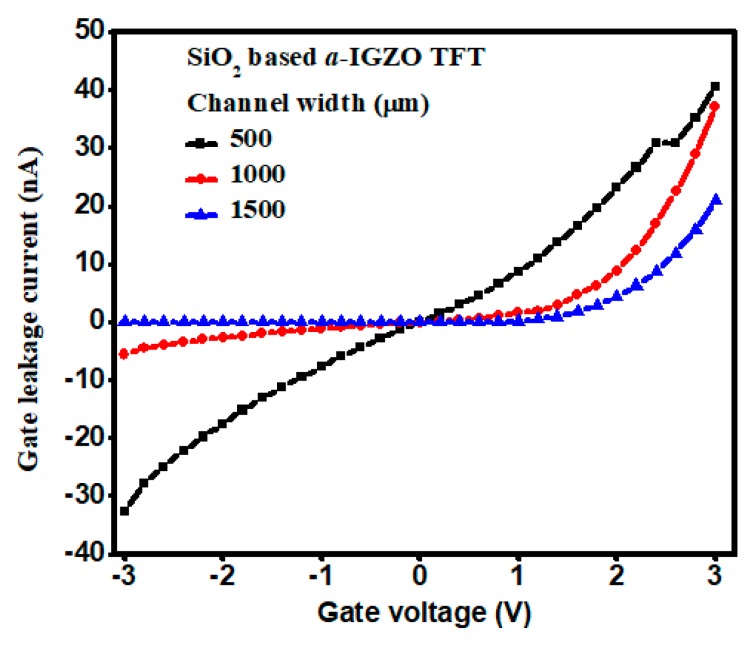
Gate leakage current for the SiO_2_-based *a*-IGZO TFTs.

It has been noted that the drain current dropped at high applied gate voltages for the low channel width TFTs. This is an indication of leakage current. The leakage current results are therefore derived and shown in Figure 7 for the three different channel widths of SiO_2_-based *a*-IGZO TFTs. The maximum gate leakage current was40.5 nA, 37.2 nA, and 20.8 nA at the gate voltage of 3V, in correspondence to the channel width of 500 µm, 1000 µm, and 1500 µm, respectively. It was thus clearly evidenced that the TFT gate leakage current would be effectively decreased with increasing channel width/length (W/L) ratio.

The threshold voltages were calculated from the extrapolation of the *I*_ds_^1/2^ vs. *V_gs_* transfer characteristic curves. The *V*_th_ values were 0.83 V, 0.73 V, and 0.48 V for the gate dielectric SiO_2_, Si_3_N_4_, and Ta_2_O_5_ samples, respectively, at the fixed channel width of 500 μm. They became 0.85 V, 0.74 V, and 0.49 V, respectively, at the channel width of 1000 μm, and 0.88 V, 0.75 V, and 0.50 V at the fixed channel width of 1500 μm. The high-k dielectric Ta_2_O_5_ and Si_3_N_4_ were shown with lower threshold voltages than the SiO_2_ for each of the fixed-channel-width *a*-IGZO TFTs. The lower power consumption application by the high-k-based *a*-IGZO TFTs would be more suitable than that by the SiO_2_ gate dielectric. However, the threshold voltage was varied along the channel width sizes at the range of 0.05 V, 0.02 V, and 0.02 V, with the corresponding gate dielectric SiO_2_, Si_3_N_4_, and Ta_2_O_5_, respectively. It should be noted that the variation of the threshold voltage along the channel width decreased with the increase in the insulating properties of dielectrics. Thus, more stable *a*-IGZO TFTs could be achieve using the high-k gate dielectrics Ta_2_O_5_ and Si_3_N_4_, instead of using SiO_2_. The threshold voltages decreased with an increased capacitance density of materials, rather than by the effect of surface roughness of gate dielectrics. The smaller variation in threshold voltages along the channel width suggested better TFT candidates with respect to stability and reliability testing. Thus, it becomes interesting to discuss the stress measurement characteristics.

**Figure 8:**
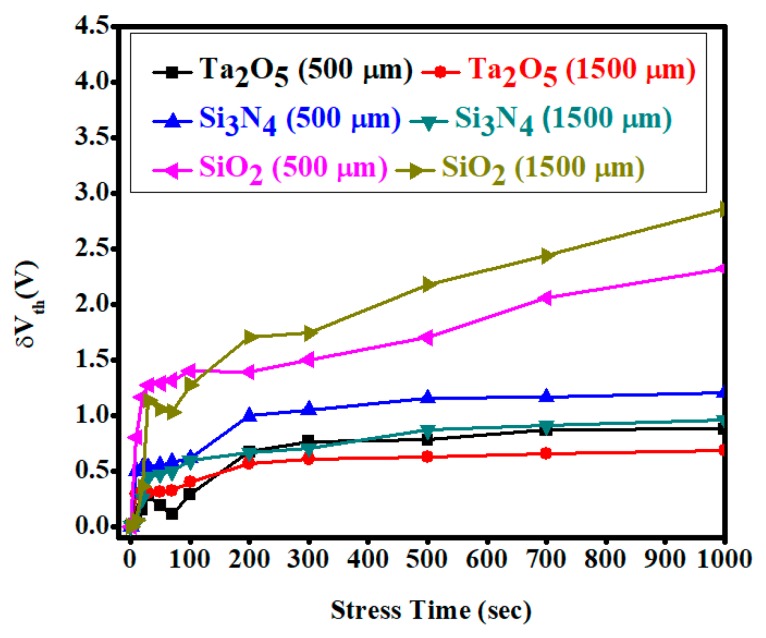
Change of threshold voltage variation under a positive bias stress (PBS) condition for 1000 s using gate dielectric SiO_2_, Si_3_N_4_, and Ta_2_O_5_ at different channel widths of 500 µm and 1500 µm. The positive stress voltage was +3 V.

The threshold voltage variation under positive bias stress (PBS) on different channel widths of 500 μm and 1500 μm with the e-beam deposited gate dielectric SiO_2_-, Si_3_N_4_-, and Ta_2_O_5_-based *a*-IGZO TFTs are displayed in Figure 8. The threshold voltage shifted towards a positive direction for all types of *a*-IGZO TFTs using the stress voltage of 3 V for 1000 s. However, the variation in the threshold voltage was much smaller for the high-k dielectric Ta_2_O_5_ samples. The threshold voltage shifted more positively in the order of Ta_2_O_5_, Si_3_N_4_, and SiO_2_ gate dielectric-based TFTs along the stress time. It should also be noted that the trapping charge density decreased with increasing the channel width of the TFTs, in accordance with the smaller threshold voltage shift for Ta_2_O_5_ and Si_3_N_4_ gate dielectric samples. The performances of the *a*-IGZO TFTs under a stress condition could be further improved with a suitable surface treatment process, such as a thermal annealing and plasma treatment process.

**Figure 9:**
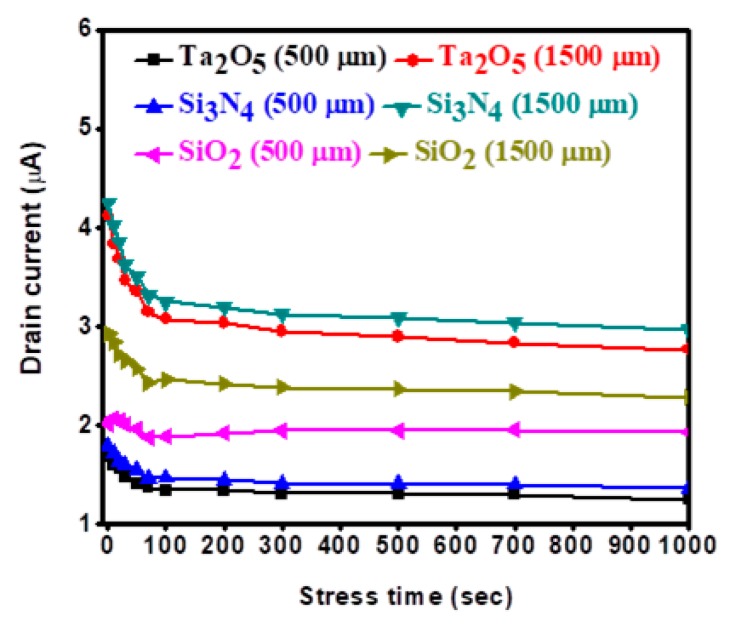
Drain current variation under a positive bias stress (PBS) condition for 1000 s with gate dielectric SiO_2_, Si_3_N_4_, and Ta_2_O_5_ at different channel widths of 500 µm and 1500 µm. The positive stress voltage was +3 V.

The drain current variation under PBS on different channel widths of 500 μm and 1500 μm with the e-beam deposited gate dielectric SiO_2_-, Si_3_N_4_-, and Ta_2_O_5_-based *a*-IGZO TFTs is shown in Figure 9. The drain current decreased initially (0–100 s) with increasing the stress current, indicating the decreased on–off current ratio for all types of *a*-IGZO TFTs under a bias stress condition. The smaller variation of drain current was associated with the lower channel width size of 500 μm for all gate dielectric TFTs. The variations were shown to be higher from SiO_2_ to Si_3_N_4_ and Ta_2_O_5_, or 0.64 µA, 1.29 µA, and 1.35 µA, respectively. The results suggested that the quantitative value of the on–off current ratio could be decreased from SiO_2_ to Si_3_N_4_ and Ta_2_O_5_ under PBS. 

The comparative study among all the e-beam deposited gate dielectric materials SiO_2_, Si_3_N_4_, and Ta_2_O_5_ exhibited potential performances in *a*-IGZO TFT application. However, the as-deposited SiO_2_ showed better basic performances in terms of mobility, current on–off ratio and sub-threshold voltages for all the dimension sizes. Nevertheless, its threshold voltage variation and threshold voltage shifting with a positive bias stress, along the channel width, was much bigger compared to those of e-beam deposited high-k gate dielectric Si_3_N_4_ and Ta_2_O_5_. The reported performances of *a*-IGZO TFTs with high-k dielectric zirconium oxide (ZrO_2_) at different channel widths were shown to be similar to the present study, where only a negligible threshold voltage variation along the channel width size was observed [21]. That is why those high-k dielectrics could still be more useful in real applications with adequate surface treatments. In the meantime, the width dependent characteristics of *a*-IGZO TFTs under stress voltage may produce the drain current variation because of the generation of a parasitic transistor, as a result of the high electric field at the insulator corner due to smaller insulator thickness [22]. Moreover, the basic, as well as the reliability performances of Si_3_N_4_-and Ta_2_O_5_-based *a*-IGZO TFTs could be further improved using some surface treatment methods to reduce the surface roughness for future applications [12].

**Figure 10:**
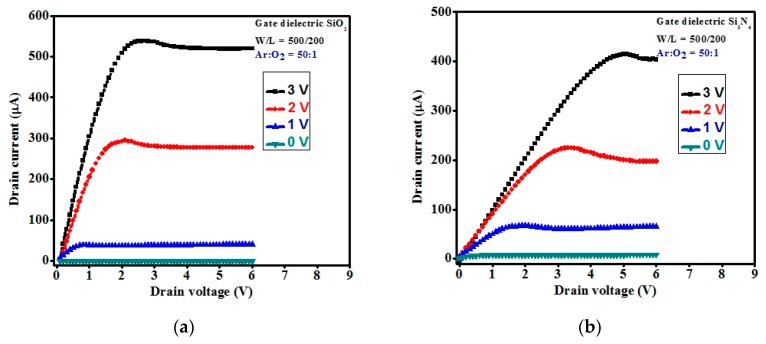

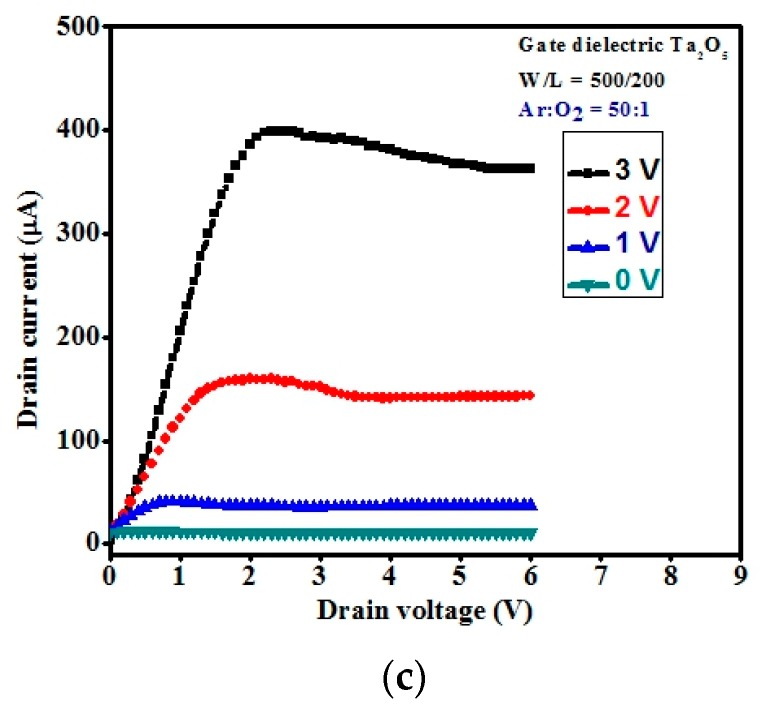
The output characteristics of the *a*-IGZO TFTs with different gate dielectric (**a**) SiO_2_, (**b**) Si_3_N_4_, and (**c**) Ta_2_O_5_ at channel width of 500 µm. The gate voltage varied from 0 V to 3 V with an increased step voltage of 1 V.

In addition, the output characteristics of the *a-*IGZO TFTs are shown in Figure 10 for the e-beam deposited gate dielectrics of SiO_2_, Si_3_N_4_, and Ta_2_O_5_. The saturation drain current was decreased in the order of SiO_2_, Si_3_N_4_, and Ta_2_O_5_, at all the applied gate voltages in the range of drain voltage of 0 to 6 V with a step voltage of 1 V. All *a*-IGZO TFTs exhibited a good operation in the n-channel enhancement mode with a low drain current at zero gate voltage. The positive gate voltage enabled the production of electrons in the channel materials. It also exhibited clear pinch-off voltages and current saturation at the higher drain voltage. The drain current was observed to increase linearly with the drain voltage at a low gate voltage. This is in agreement with creating an ohmic contact of the source and drain. It has been proven that the TFTs met the standard field-effect transistor characteristics. Additionally, the decreased drain current at particular gate and drain voltages showed similar trends with the transfer characteristics of the *a*-IGZO TFTs with all three gate dielectrics along the channel width. The better ohmic contact achieved with gate dielectric SiO_2_ was due to the good interface contact of source and drain with the channel material surface, because smoother surface provided a lower parasitic effect [17]. On the other hand, the non-idealities in the output characteristics and a slightly negative degradation or hump effect were observed between the pinch-off and saturation regions. This phenomenon became more prominent with Ta_2_O_5_-based *a*-IGZO TFTs, likely due to interfacial resistance between layers. An additional surface treatment process for the gate dielectric layer could reduce this effect and achieve better ohmic contact.

## 4. Conclusions

The performances of *a*-IGZO TFTs have been very much dependent upon the insulting properties of gate dielectric materials and the surface roughness. The e-beam as-deposited SiO_2_ presented the best basic electrical performances with a mobility of 27.7 cm^2^/V·s, 21.8 cm^2^/V·s, and 13.3 cm^2^/V·s along different channel width sizes of 500 μm, 1000 μm, and 1500 μm, respectively, because of the smoother surface properties. However, the reliability in terms of stress measurement revealed that the better stability with a smaller change in threshold voltage along the channel width should be achieved with an increased capacitance density of film, corresponding to the e-beam deposited gate dielectric Ta_2_O_5_. In addition, the results suggested that the low-cost, simple processing by an e-beam evaporator for depositing dielectric material SiO_2_, Si_3_N_4_, and Ta_2_O_5_ could be used to obtain high-performance *a*-IGZO TFT devices. Special treatment on the surface to reduce surface roughness can further enhance the insulating properties. The channel width-dependent electrical performances for all types of *a*-IGZO TFTs clarified the threshold voltage variation at the room-temperature fabrication process with the SiO_2_ gate dielectric. This phenomenon could be avoided by replacing with high-k dielectrics, such as Si_3_N_4_ and Ta_2_O_5_, where threshold voltage variation is much smaller compared tothat of SiO_2_. Therefore, this investigation of channel width-dependent electrical properties with different gate dielectrics should be applied for future development to design higher-performance *a*-IGZO TFTs.

## Figures and Tables

**Table 1:** The performance parameters of *a*-IGZO TFTs with SiO_2_ gate dielectric.

Width (μm)	*V*_th_ (V)	*I*_on_/*I*_off_	*SS* (V/dec)	*µ*_fet_ (cm^2^/V·s)
500	0.83	2.9 × 10^7^	0.11	27.9
1000	0.85	4.6 × 10^7^	0.11	21.6
1500	0.88	8.1 × 10^7^	0.10	13.5

**Table 2:** The performance parameters of *a*-IGZO TFTs with Si_3_N_4_ gate dielectric.

Width (μm)	*V*_th_ (V)	*I*_on_/*I*_off_	*SS* (V/dec)	*µ*_fet_ (cm^2^/V·s)
500	0.73	1.5 × 10^5^	0.15	20.6
1000	0.74	2.1 × 10^6^	0.13	13.5
1500	0.75	7.1 × 10^6^	0.11	8.8

**Table 3:** The performance parameters of *a*-IGZO TFTs with Ta_2_O_5_ gate dielectric.

Width (μm)	*V*_th_ (V)	*I*_on_/*I*_off_	*SS* (V/dec)	*µ*_fet_ (cm^2^/V·s)
500	0.48	7.1 × 10^5^	0.14	12.1
1000	0.49	2.1 × 10^5^	0.11	6.4
1500	0.50	1.6 × 10^6^	0.10	4.7
